# Supplementary material for: Superplastic nanoscale pore shaping by ion irradiation
Source: Nat Commun. 2018 Feb 26;9:835. doi: 10.1038/s41467-018-03316-7 (PMC5827561; doi:10.1038/s41467-018-03316-7)
Supplement: Supplementary file 1 — Supplementary Information [file 41467_2018_3316_MOESM1_ESM.pdf]

Supplementary Information for

Superplastic nanoscale pore shaping by ion irradiation

Morteza Aramesh* [1,2,3], Yashar Mayamei[4], Annalena Wolff[5], and Kostya (Ken) Ostrikov[1,2]

1. *School of Physics, Chemistry and Mechanical Engineering and Institute for Future Environments, Queensland University of Technology (QUT), Brisbane QLD 4000, Australia*
2. *CSIRO-QUT Joint Sustainable Processes and Devices Laboratory, Commonwealth Scientific and Industrial Research Organisation, Lindfield NSW 2070, Australia*
3. *Laboratory of Biosensors and Bioelectronics, Institute for Biomedical Engineering, ETH Zürich, Zürich 8092, Switzerland*
4. *Department of Nano Science, University of Science and Technology, Daejeon 34113, Republic of Korea*
5. *Central Analytical Research Facility, Institute for Future Environments, Queensland University of Technology (QUT), Brisbane QLD 4000, Australia*

** Corresponding author: Morteza Aramesh, Email: maramesh@ethz.ch; mrtz.aramesh@gmail.com*

Supplementary Methods:

AAO thin film preparation

The samples were prepared by two-step anodization.[1] Briefly, pure aluminum discs (99.999% purity) in 2 cm diameter and 1 mm thickness were washed by deionized water and then were electropolished in a 1:3 mixture of $HClO_4$ (65 %vol) and ethanol (99.6 %vol) with gentle stirring at 1ºC. The electropolished samples were placed in an electrochemical cell with anodization area of 0.95 cm^2 and the first anodization was performed in a solution of 0.3 M oxalic acid at 1ºC by applying 40 V for 24 hours. Then the formed porous layer was removed by a mixture of H_3PO_4 (6 %wt) and H_2CrO_4 (1.8 %wt). Second anodization was performed with the same condition as first-step for 500 sec. To transfer the thin AAO films on silicon substrates, first a protective layer of Polystyrene (in chloroform 1.5:10) was spin coated on surface of the thin-film at 2000 rpm, followed by 2 min annealing at 60 ºC on a hotplate. The remaining aluminum on the back of the oxide layer was removed in a diluted solution of $CuCl_2$:HCl (~ 2 hours at 1ºC). The floating samples were transferred (with a use of glass slides) to another petri-dish containing nitric acids to remove the residual copper. This step was followed by another transfer to a DI-water containing petri-dish to clean the surface. The barrier-layer in the oxide layer was removed by transferring the films to a solution of phosphoric acid (5 %wt) at 30 ºC for 35 min. After this step the samples were transferred three times to DI water solutions. Crystalline silicon substrates with (100) crystal orientation were used to take out the membranes from water. The protective Polystyrene films were removed by a chloroform solution and the membranes were washed with copious amount of water and then dried under a gentle Nitrogen flow. One week after the preparation process (for dehydration), surface of the samples were cleaned by exposure to oxygen plasma (100 W for 5 min) immediately before being transferred to HIM for ion-matter interaction studies.

Crystalline AAO

Crystalline AAO were obtained by annealing the membranes at 800 ºC in a furnace with a flow of Ar at 0.5 Torr for 8 hours. The resulted crystalline structure in similar conditions has been shown elsewhere.[2]

Ion Beam irradiation

Helium-Ion-Microscope (Zeiss ORION NanoFab or Zeiss Orion Plus, depend on the availability) was used in this study. 5-45 keV Helium ions were generated and accelerated at GFIS and were delivered to the sample through a 10 μm aperture. The ion beam current was initially within 0.1–10 pA range (depending on the ion energy). The gas ionization apparatus was housed in a high-vacuum chamber within the range of 10^{-7}-10^{-5} mbar and the ion beam flux (ions.$nm^{-2}sec^{-1}$) was controlled by tuning the chamber pressure with 0.1 pA precision.

The experiments were mainly performed at room-temperature. Some experiments (mentioned in the main text) were performed at higher temperatures by pre-heating the substrate before irradiation. Samples were heated in the preloading chamber just before putting the sample into the main chamber for immediate analysis. The temperature in the preloading chamber was measured with an IR thermometer. The sample temperature inside the main chamber is expected to be within 10 °C of the quoted temperature. Beam alignment and focus was optimized at spots proximal to the region of interest before each measurement. The ion-beam was swept over the sample with dwelling time of 0.1 μs. The results for the dwelling time effects are not presented in this study, but it was found that 0.1 μs is the optimum value to achieve maximum consistency in mass-flow patterns. The live images were recorded at 50 Hz for image analysis of the closing pores and then the temporal pore size shrinkages were measured using ImageJ software.

Electron Microscopy Analysis

The FEI Scios FIB/SEM was used to prepare TEM lamellas of the irradiated sample areas. To protect the sample surface from any ion irradiation, the areas of interest were first covered with a protective electron beam deposited platinum strip using 2 kV acceleration voltage and 3.2 nA electron beam current, followed by an ion beam deposited platinum strip with 30 kV acceleration voltage and an ion beam current density of 6 $pA.\mu m^{-2}$. The cross-sections were cut using the following gallium ion beam parameters: 30 kV and 5 nA ion beam current. The lamella was thinned at 30 kV using 0.5 nA and followed by 0.1 nA ion beam current. The thin lamella was subsequently polished with 5 kV, 16 pA and 2 kV, 8.9 pA. HRTEM/TEM analysis was carried out in a FEI Tecnai TF20 HRTEM (equipped with an electron spectrometer for EELS) and Jeol 2100 TEM 200 kV systems. ImageJ was used to apply colours in EELS images.

Stopping and Range of Ions in Matter (SRIM): Monte Carlo simulations

The stopping range/penetration depth of the He ions at different voltages was predicted by computer simulation program SRIM (Stopping and Range of Ions in Matter)[3]. 10000 ions were simulated using the monolayer collision steps calculation type. The following parameters was used for simulations: 100 nm-thick aluminium oxide film (60% O and 40% Al) on top of a 1000 nm-thick Si layer. The displacement energy of the atoms were 20, 25 and 15 eV for O, Al and Si, respectively.

Supplementary Figures:

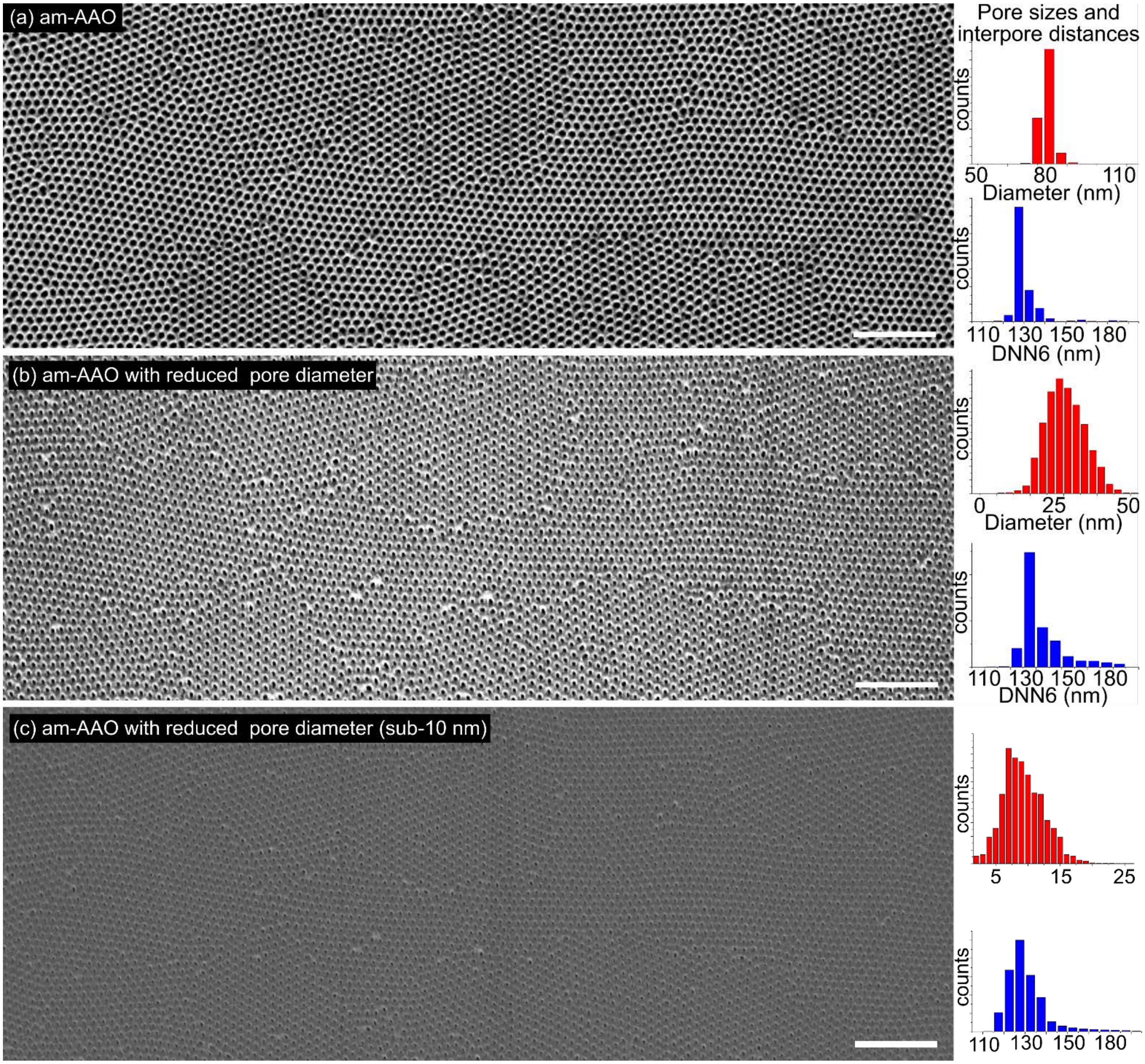

Supplementary Figure 1: Large-area HIM micrographs of (a) non-irradiated am-AAO and (b) irradiated am-AAO with 25 keV helium ions at the low-flux regime: F=10 ions.nm^{-2}s^{-1}. (a) The average pore size of the non-irradiated pores was 78±4 nm. The irradiated am-AAO (b,c) exhibit smaller pore sizes as well as a broadened interpore distances, evident in the HIM micrographs and the corresponding size distributions. The average pore diameter was 27±5 and 7±5 nm after irradiation with a fluence of ~5×10^{14} and ~2×10^{15} ions.cm^{-2}, respectively. The scale bar is 1 µm in (a-c). DNN6 is the average distance between six nearest neighbouring pores, and is used as a measure of average interpore distance.

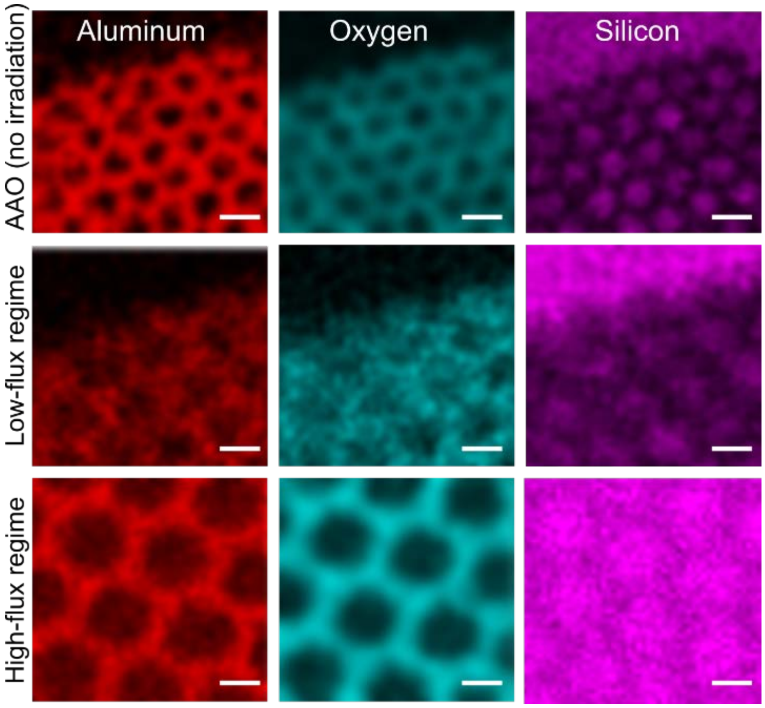

Supplementary Figure 2: Chemical analysis: SEM EDX of am-AAO membranes before and after irradiation with 25 keV He^+ at different flux regimes (F=10 and 100 ions.nm^{-2}s^{-1} for the low and high flux regimes, respectively). In all of the cases the three major chemical elements are Aluminum, Oxygen and Silicon (substrate). The scale bar is 100 nm. *n.b.* Spatial resolution of EDX is around 2 µm and therefore it is not possible to completely separate the signal from Si substrate and alumina superstrate. Please refer to Figure 3 of the manuscript for high-resolution TEM analysis and EELS maps.

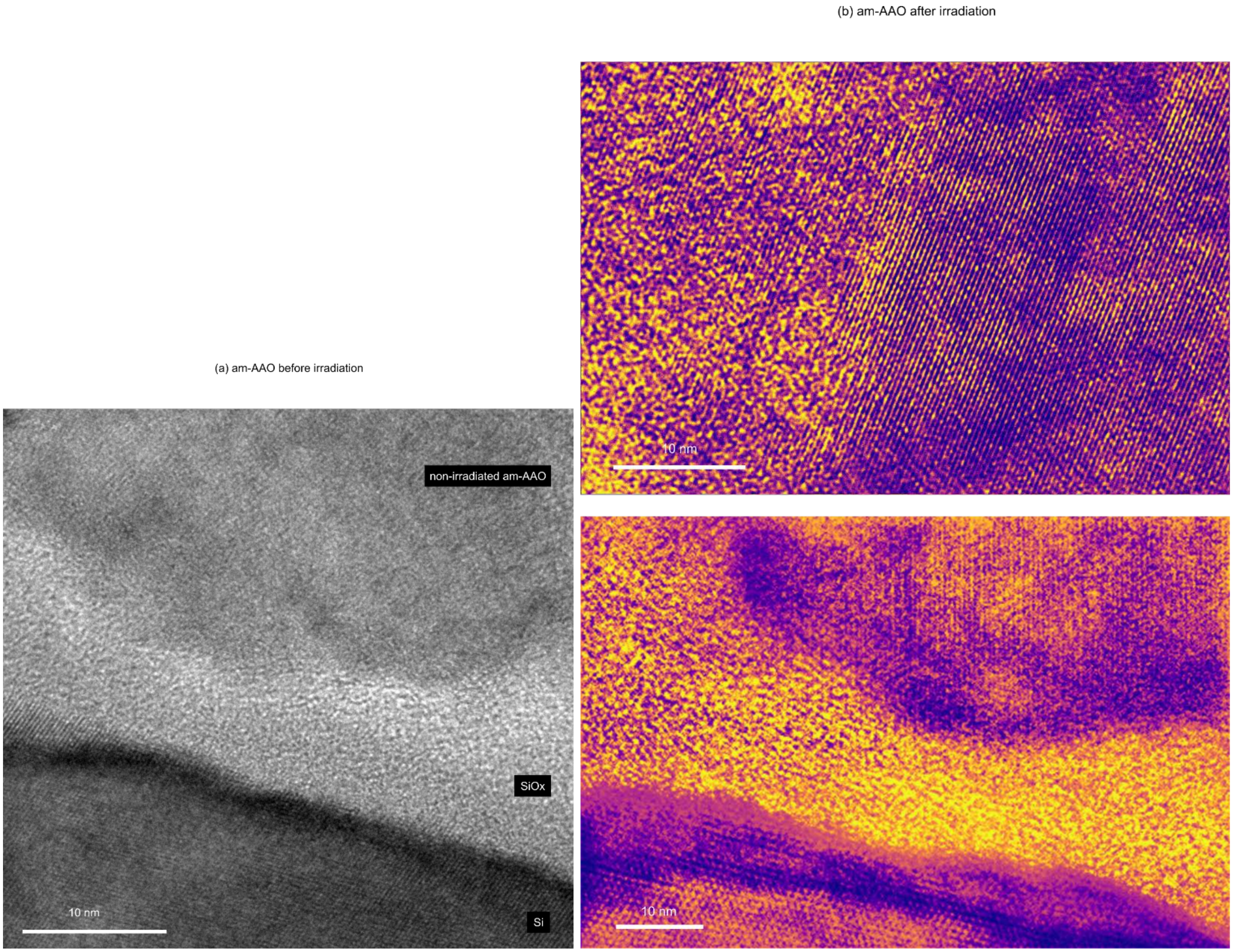

Supplementary Figure 3: (a) Atomic structure of am-AAO in HRTEM before irradiation, indicating that the as-prepared AAO membranes (non-irradiated) are amorphous in their atomic structure. (b) Crystallization of am-AAO with He^{+} irradiation at 25 keV , clear from the lattice fringes.

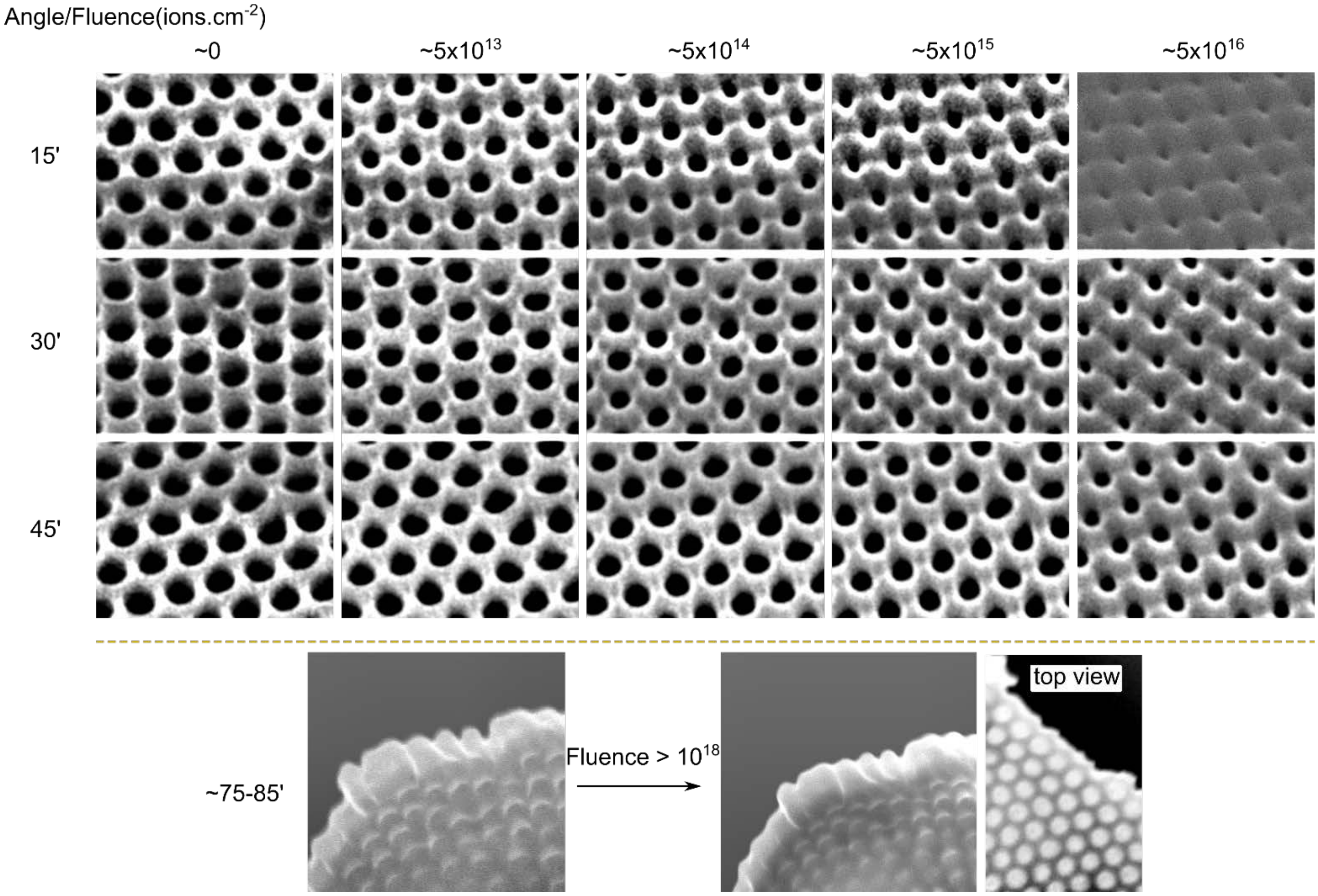

Supplementary Figure 4: Angle dependence: Irradiation of the sample (within the low-flux regime) was performed at different incident angles of ions. Generally the efficiency of pore closure was reduced due to increasing of the incident angle. An experiment on free-standing membranes at high angle of incidence (estimated to be > 75°) indicated that the efficiency of pore closure significantly reduces at very high angles. In the image below, a free-standing membranes was irradiated with > 10^{18} ions.cm^{-2}, however no significant reduction in pore size was realized. The top view of the pores after irradiation is shown in the same figure (the brightness of the pores is most probably due to the charging effect).

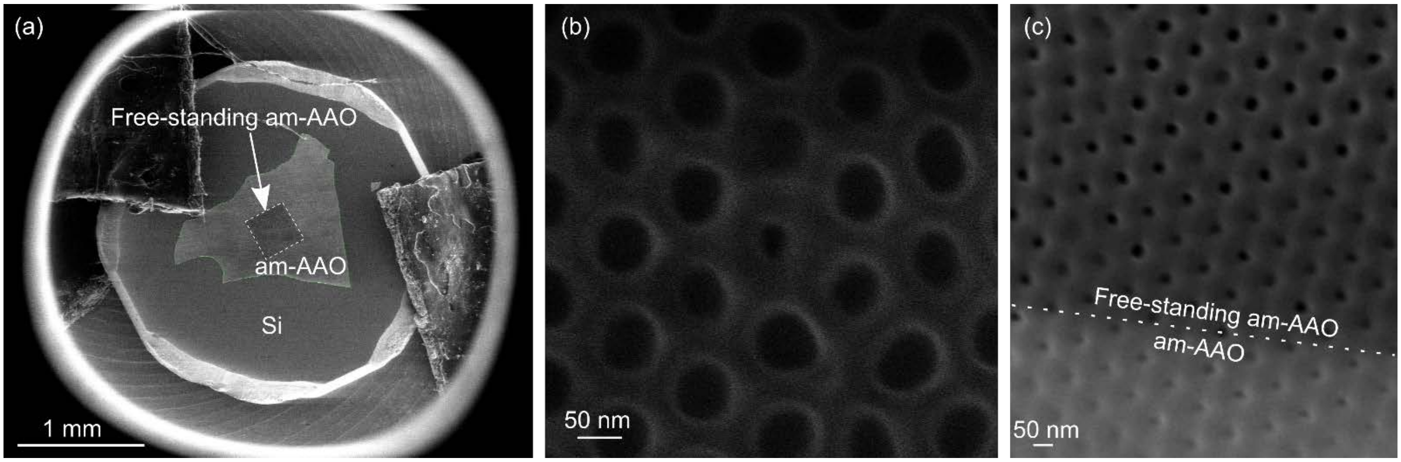

Supplementary Figure 5: Free-standing membranes. (a) am-AAO membranes were transferred on a substrate with a rectangular hole in the middle (50 µm×50 µm). The hole was made by selectively etching a thin film SiN membrane (10 nm-thick) on top of a Si in a standard TEM grid (item # NT005Z from Norcada). (b) Demonstration of single-pore shrinkage in a free-standing part of the membrane. (c) shows the border line between the free-standing and supported area after irradiation with (low-flux) 25 keV He^+. Both area exhibit pore shrinkage.

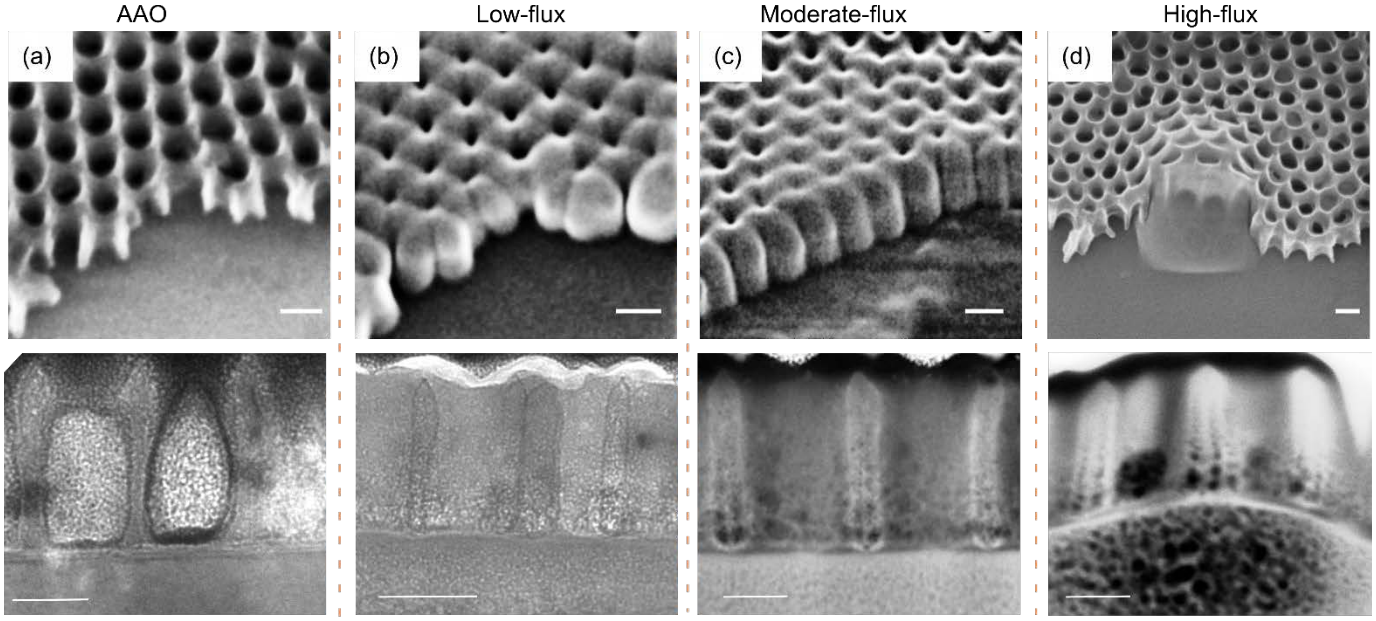

Supplementary Figure 6: HIM cross-sectional views of am-AAO before and after irradiation at different flux regimes. The non-irradiated am-AAO is displayed in (a). At low-flux regime (b), in addition to shrinkage of the pores, the thickness of the membrane reduced after irradiation (fluence: ~10^{15} ions.cm^{-2}). In moderate-flux regime (c), the pores exhibit a reduced pore diameter, however, the thickness of the AAO layer was slightly increased (fluence: ~5×10^{16} ions.cm^{-2}). At high-flux regime (d), pore size do not shrink (fluence: ~10^{18} ions.cm^{-2}). The scale bar in HIM image is 100 nm. The cross-sectional TEM images from the irradiated am-AAO at different flux regimes are shown below of each image. Formation of nanobubbles (cavities) in irradiated am-AAO is evident in TEM images. (*n.b.* for the TEM images the estimated fluence from left to right is: ~10^{15} ions.cm^{-2} , ~10^{16} ions.cm^{-2} and ~10^{17} ions.cm^{-2}). The scale bar in TEM image is 50 nm.

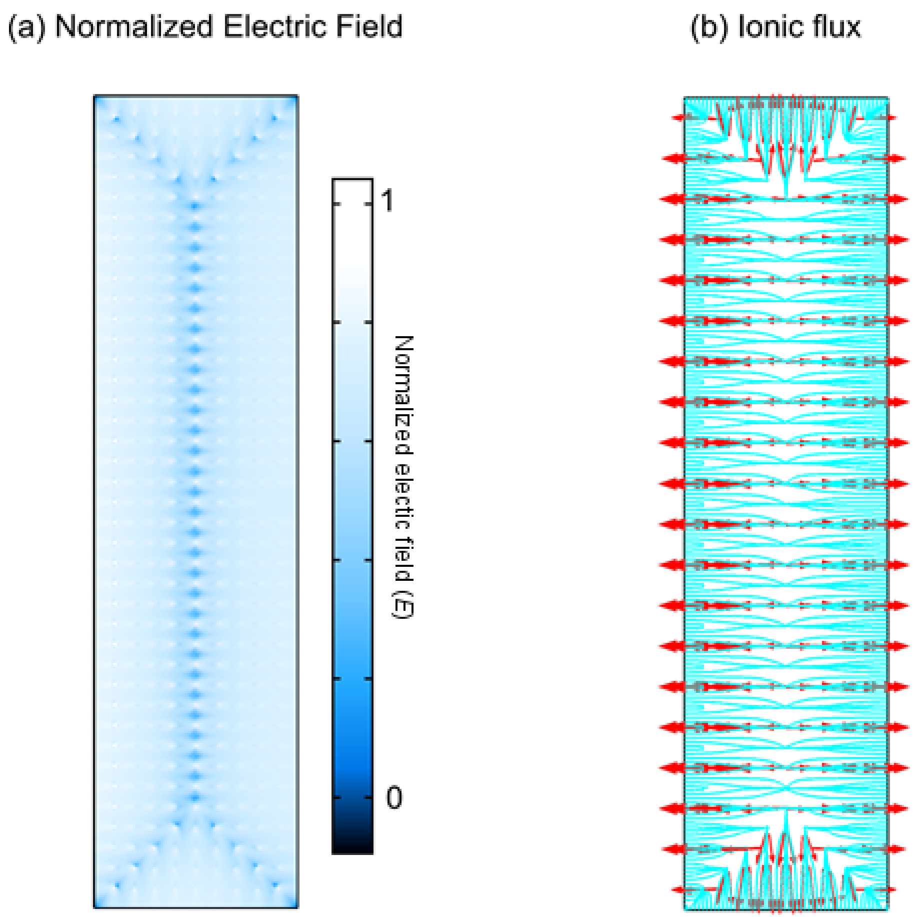

Supplementary Figure 7: FEM of ionic flow in an ionized network. The Finite-element-method simulations (FEM) is based on simple concept of ionic flow (i.e. Al^{3+} and O^{2-}) inside of a boundary with an ionized network of atoms. The ionized network of the atoms is simulated by setting point charges in a rectangular array within the boundary with arbitrary distances from each other (for simplicity an array of 12×42 points are demonstrated here – Figure S7.a shows the normalized electric field in such a network). By approximating the oxide as a Newtonian fluid and solving Navier-Stokes and continuity equations, the migration flux of ionic species (termed as ionic flux) induced by the electric fields is obtained (Figure 7S.b). The dominance of the ionic flux around the pore walls is evident from this figure. Higher ionic flux at the central edges is consistent with the experimental observation and TEM images in Figure 2 of the manuscript.

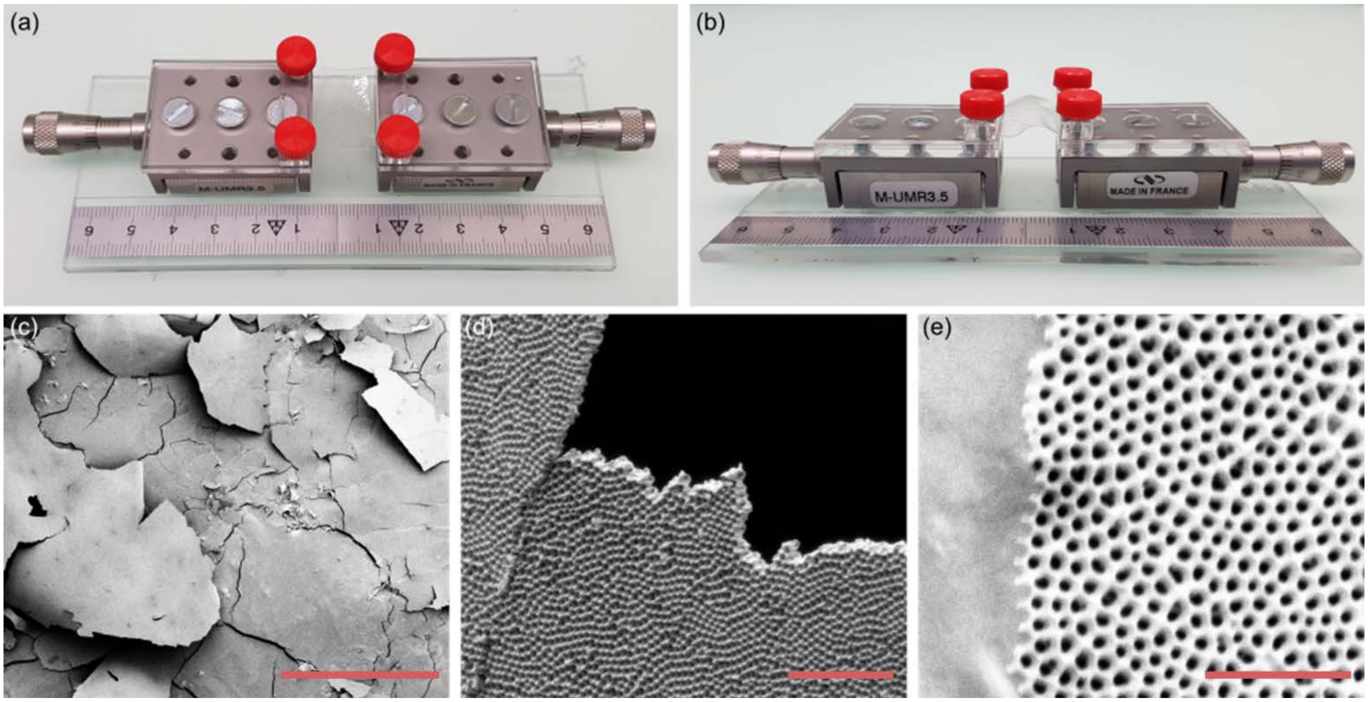

Supplementary Figure 8: Elasticity of thin films of am-AAO. Stretching and bending experiments were performed on thin film am-AAO membranes. For this purpose, first thin films of am-AAO (100 nm-thick) were transferred on semi-cured PDMS elastomers. The am-AAO on PDMS then were mounted on stretching device, as shown in (a). The bending experiment was performed as shown in (b). (c-e) show typical SEM images of the samples after stretching/bending experiment, with significant damages on the samples. The broken boundaries exhibit sharp cuts both in nano- and micro-scales, with no indication of plastic deformation around the edges. It seems that the am-AAO membranes break very easily under moderate stretching (<5%) or bending constrains. The scale bars are 50, 5 and 3 μm for (c), (d) and (e), respectively.

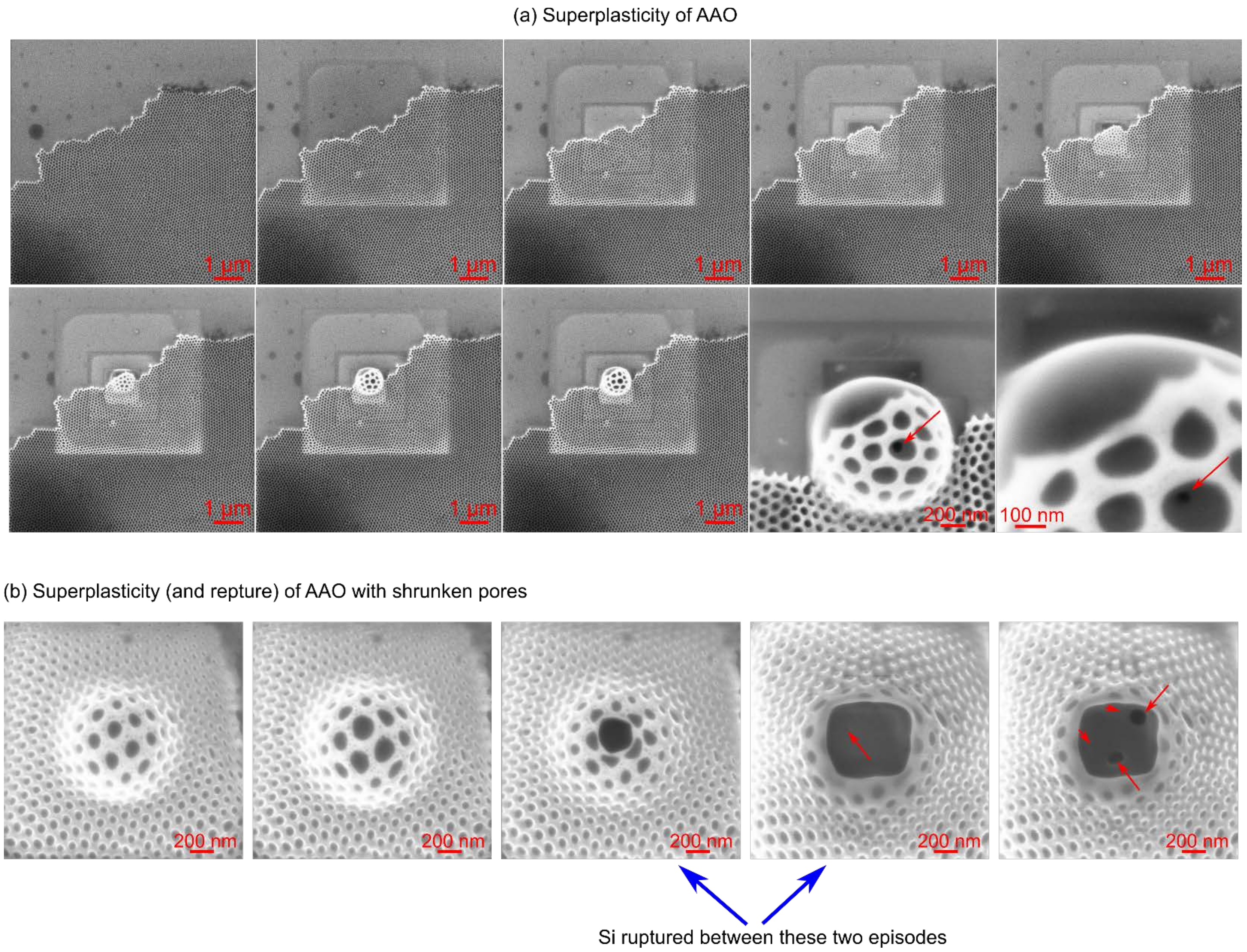

Supplementary Figure 9: Superplasticity and rupture of am-AAO : (a) am-AAO exhibits superplasticity when irradiated He^{+} ion beams (images were taken after irradiation at 25 keV), by being able to take uniform morphological changes more than 200% of the original structure (area of the pores can be used as a measure for expansion). Am-AAO did not rupture under extreme tension induced by the swollen substrate. At high fluences (>10^{21} ions.cm^{-2}), the Si substrates ruptures and small holes start to appear on the surface (shown by red arrows). (b) shows that am-AAO with shrunken pores is also able to be take controlled superplastic deformations. The rupture of Si substrate is evident between two episodes of these series, where the substrate collapse. Further irradiation continues to make extra holes in the collapsed Si structure, but am-AAO remains within its plastic deformation.

Supplementary References:

1- Aramesh, M. Djalalian-Assl, A. Aghili Yajadda, M. Prawer, S. Ostrikov, K. Thin Nanoporous Metal–Insulator–Metal Membranes. *ACS Applied Materials & Interfaces* **8**, 4292-4297 (2016)

2- Aramesh, M. *et al.* Multifunctional three-dimensional nanodiamond-nanoporous alumina nanoarchitectures. *Carbon* **75**, 452-464 (2014).

3- Ziegler, J. F., Ziegler, M. D. & Biersack, J. P. SRIM–The stopping and range of ions in matter. *Nuclear Instruments and Methods in Physics Research Section B: Beam Interactions with Materials and Atoms* **268**, 1818-1823 (2010).
