# Supplementary material for: Superplastic nanoscale pore shaping by ion irradiation
Source: Nat Commun. 2018 Feb 26;9:835. doi: 10.1038/s41467-018-03316-7 (PMC5827561; doi:10.1038/s41467-018-03316-7)
Supplement: Supplementary file 3 — Description of Additional Supplementary Files [file 41467_2018_3316_MOESM3_ESM.pdf]

Description of Additional Supplementary Files

File Name: Supplementary Movie 1
Description: AAO pores shrinkage at low-flux irradiation - 25keV He+.
